# Designing a Healthy Food Partnership: lessons from the Australian Food and Health Dialogue

**DOI:** 10.1186/s12889-016-3302-8

**Published:** 2016-07-27

**Authors:** Alexandra Jones, Roger Magnusson, Boyd Swinburn, Jacqui Webster, Amanda Wood, Gary Sacks, Bruce Neal

**Affiliations:** 1 The George Institute for Global Health, Level 10, KGV Building, Missenden Rd, Camperdown, NSW 2050 Australia; 2 The Charles Perkins Centre, University of Sydney, Sydney, Australia; 3 Sydney Law School, University of Sydney, Sydney, Australia; 4 University of Auckland, Auckland, New Zealand; 5 WHO Collaborating Centre for Obesity Prevention, Deakin University, Melbourne, Australia; 6 WHO Collaborating Centre on Population Salt Reduction, The George Institute for Global Health, Sydney, Australia

## Abstract

**Background:**

Poor diets are a leading cause of disease burden worldwide. In Australia, the Federal Government established the Food and Health Dialogue (the Dialogue) in 2009 to address this issue, primarily through food reformulation. We evaluated the Dialogue’s performance over its 6 years of operation and used these findings to develop recommendations for the success of the new Healthy Food Partnership.

**Methods:**

We used information from the Dialogue website, media releases, communiqués, e-newsletters, materials released under freedom-of-information, and Parliamentary Hansard to evaluate the Dialogue’s achievements from October 2013 to November 2015, using the RE-AIM (reach, efficacy, adoption, implementation and maintenance) framework. We also engaged closely with two former Dialogue members. Our findings update a prior assessment done in October 2013.

**Results:**

Little data is available to evaluate the Dialogue’s recent achievements, with no information about progress against milestones released since October 2013. In the last 2 years, only one additional set of sodium reduction targets (cheese) was agreed and Quick Service Restaurant foods were added as an area for action. Some activity was identified in 12 of a possible 137 (9 %) areas of action within the Dialogue’s mandate. Independent evaluation found targets were partially achieved in some food categories, with substantial variation in success between companies. No effects on the knowledge, behaviours or nutrient intake of the Australian population or evidence of impact on diet-related disease could be identified.

**Conclusions:**

The new Healthy Food Partnership has similar goals to the Dialogue. While highly laudable and recognised globally as cost-effective, the mechanism for delivery in Australia has been woefully inadequate. Strong government leadership, adequate funding, clear targets and timelines, management of conflict of interest, comprehensive monitoring and evaluation, and a plan for responsive regulation in the event of missed milestones will be required if the new Healthy Food Partnership is to achieve its urgent public health goals.

## Background

Chronic, non-communicable diseases (NCDs) are the main causes of premature death and disability in Australia [1], and around the world [2]. Dietary risk factors – including diets high in salt, saturated fats, added sugar and energy, with inadequate intake of fruits, vegetables and wholegrains – are now the leading cause of this disease burden in Australia, driving epidemics of high blood pressure, diabetes, obesity, vascular disease and cancer [3, 4].

Acknowledging the need to assist Australians to modify poor dietary habits, the Federal Government established the Food and Health Dialogue (the Dialogue) in March 2009. Similar public-private partnerships implemented elsewhere include the United Kingdom’s ‘Public Health Responsibility Deal’ [5] and the ‘National Sodium Reduction Initiative’ in the United States [6]. Specified objectives were to raise “the nutritional profile of foods through reformulation, consumer education and portion standardization” and to provide “a framework for government, public health groups and industry to work collaboratively across all levels of the food supply chain to improve dietary intakes.” Between 2013 and 2015 the Dialogue process apparently lapsed. In November 2015, the government announced a successor to the Dialogue, the ‘Healthy Food Partnership.’ Details of the Healthy Food Partnership are scant but the high level objectives appear broadly similar [7].

We previously carried out a systematic interim assessment of the extent to which the Dialogue delivered upon its objectives over the first 4 years of operation [8]. We found that the Dialogue had laudable goals although the mechanism for delivering them was inadequate. We made recommendations for strengthening the Dialogue’s effectiveness and accountability, including agreement on more explicit objectives, the need for measurable targets, improved monitoring, evaluation and reporting, new mechanisms for enforcement, and commitment to iterative modification and periodic review of targets in response to progress made. Two years on, with the Dialogue now effectively terminated, we seek to provide an overall appraisal of the Dialogue and to identify structural criteria that will ensure the success of the new Healthy Food Partnership.

## Method

As with our interim assessment, we have adopted the RE-AIM (reach, efficacy, adoption, implementation and maintenance) framework to make our assessment of the Dialogue. This method has been widely used to evaluate the public health impact of prevention programs and health policies [9, 10].

Areas for Dialogue action were derived by multiplying 25 defined major food categories by eight possible action areas (reformulation of up to 7 nutrients and portion size) and subtracting combinations where no target was applicable (Table 1). We identified 137 areas of possible Dialogue action and sought evidence for activity in each. In addition we sought evidence of the broader Dialogue objectives of delivering consumer education and providing a framework for government, public health groups and industry to work collaboratively across all levels of the food supply chain to improve dietary intakes.

**Table 1:**
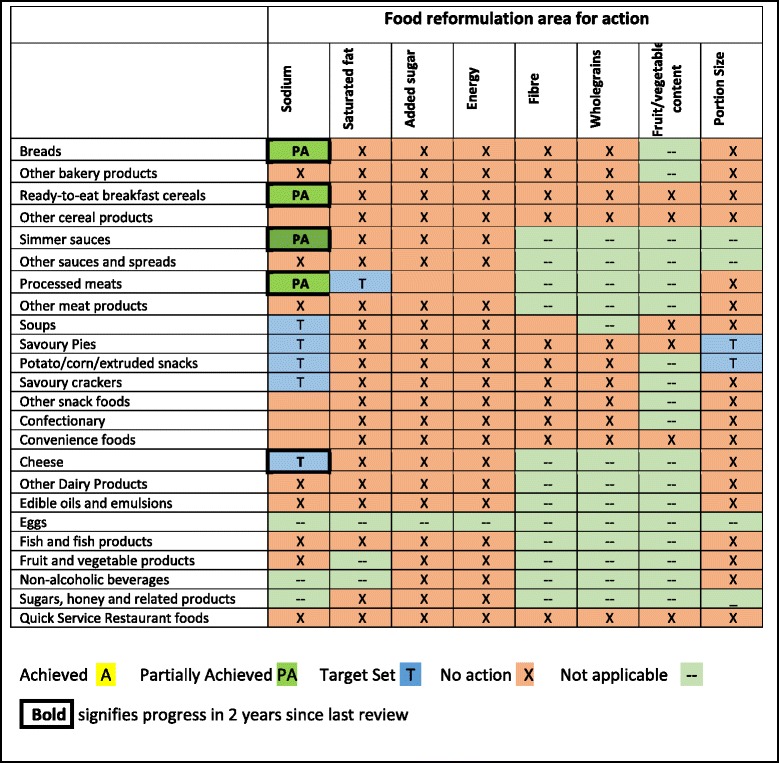
Status of Food and Health Dialogue actions on food reformulation and portion size standardization areas identified 6 years after inception

We carried out a systematic search for information about the Dialogue’s action areas, and progress towards its other objectives, between October 2013 and October 2015. We extracted information from materials published on the Dialogue website which functioned as the central repository for official communications on Dialogue activities, including media releases, communiqués and e-newsletters. This was supplemented by a systematic search of Hansard, the Department of Health’s Freedom of Information Disclosure Log, websites of participating companies, and the scientific and grey literature. Searches of websites were done using the terms ‘Food and Health Dialogue’ and ‘FHD’ through Google Chrome and Internet Explorer Browsers and reviewing the first 100 listings. The same search terms were used for Parliamentary Hansard.

We examined these materials to identify indications of intent, actions and outcomes which were grouped and summarised in terms of the REAIM dimensions. We then compared the findings for the latest 2 year period against our assessment from September 2013, taking into account other government and industry initiatives to address diet-related disease. Where possible we updated and summarised these metrics in tabular format. Since it appears the Dialogue will now be replaced, we analysed our findings to identify strategies for strengthening the design and performance of the new Healthy Food Partnership.

## Results

Available data with which to evaluate the Dialogue were limited. The Dialogue website provided no systematic reporting about ongoing activity or progress towards planned outcomes as specified by the Dialogue’s terms. The most recent e-newsletter from the Dialogue was released in November 2012 and the last known Dialogue website update occurred in July 2014. Limited additional data was obtained through Parliamentary Hansard and under Freedom of Information Disclosure. Personal communications with Dialogue participants confirm no further information is publicly available.

### Identified goals of the dialogue

The expressed goals of the Dialogue did not change throughout the life of the initiative (Table 2). The Dialogue’s primary activity was voluntary reformulation across commonly consumed food products with the aim of reducing levels of saturated fat, added sugar, sodium and energy and increasing fibre, wholegrain, fruit and vegetable content [11]. During parliamentary questioning in June 2014, Senator Fiona Nash suggested “the Dialogue could be a little more broad in what it is doing”, indicating improvements were being considered and inferred that these would be “finalised in the not-too-distant future” [12]. These improvements will presumably be reflected in the Healthy Food Partnership.

**Table 2:** Comparison of Food and Health Dialogue and Healthy Food Partnership

	Dialogue	Healthy food partnership
Overarching problem	Diet-related ill health is the leading cause of disease in Australia	Diet-related ill health is the leading cause of disease in Australia
Specified high level goals	Raise the nutritional profile of food through food innovation	Cooperatively tackle obesity and encourage healthy eating
	Provide a framework for government, public health groups and industry to work to improve the diet of the population	Work together on strategies to educate consumers on consuming fresh produce, appropriate portion sizes, and to accelerate efforts to reformulate food to make it healthier
		Complement the Health Star Rating System
Implementation strategy	Government, industry and other stakeholders	Government, industry and other (limited) stakeholders
	Operationalised through an Executive Group, working groups and industry roundtables	Operational approach currently unspecified
Chief activities	Food reformulation to agreed targets	Consumer education
	Consumer education	Portion standardisation
	Portion standardisation	Acceleration of efforts to reformulate food
Objectives	Short-term	To be specified
	• Improved composition of foods	
	• Enhanced consumer knowledge	
	• Standardised portion sizes	
	Medium-term	
	• Improved healthiness of food eaten	
	• Improved blood pressure, obesity, diabetes, blood lipids	
	Long-term	
	• Reduced burden cardiovascular diseases, cancers, musculoskeletal diseases and diabetes	
Membership of Executive	Federal Health Ministerial representative, Australian Food and Grocery, National Heart Foundation of Australia, Woolworths Ltd, Senior Dietitian and Research Scientist, Commonwealth Scientific and Industrial Research Organisation, Public Health Association of Australia, South Australia Health, McDonald’s Australia, Food Standards Australia New Zealand	Federal Health Ministerial Representative, Australian Food and Grocery Council, National Heart Foundation of Australia, Woolworths Ltd, Metcash, Coles, Public Health Association of Australia, Dietitians Association of Australia, AusVeg, Dairy Australia, Meat and Livestock Australia

### Reach and efficacy

We assessed the Dialogue’s reach and efficacy in terms of the extent to which the initiative’s actions have affected the Australian population as a whole, and delivered the intended outcomes. The Dialogue itself has released no information on the extent to which the Australian population has obtained access to reformulated foods, foods of standardised portion size, or nutrition education in the review period. No estimates of the extent to which exposure to reformulated foods and education has affected purchasing patterns, immediate physiological parameters, or measures of diet-related disease burden have been publicly released.

### Adoption and implementation

The Dialogue’s official governance structure and list of engaged companies as reported through the Dialogue website did not change during the period of review. In October 2013, the Reformulation Working Group added one further target - reducing the sodium content of cheese – bringing the total to 12 out of a possible 137 action areas (Table 1). Processed poultry, noodles and condiments were identified as the next categories to be addressed but no evidence of any action was identified. Of the twelve targets set, nine related to sodium reduction, one to saturated fat, and two to portion size. No action was taken within any food category to address reduction of added sugar or energy density, or in relation to adding under-consumed nutrients.

Sodium reduction targets for bread, ready-to-eat breakfast cereals, simmer sauces, processed meats, soups and savoury pies were due to be met between December 2013 and December 2014 but the Dialogue released no objective information as to whether these were achieved through either its website or other government communications. Published independent evaluation of breads, cereals, processed meats and pasta sauces revealed some progress, but noted that targets were only partially achieved and that there was substantial variation in success between food companies [13, 14]. In addition, targets set for some food categories were comparatively weak, e.g. the Dialogue targeted a 15 % sodium reduction in sauces containing more than 420 mg/100 g between 2011 and 2014, compared to the UK’s 2012 target of a mean sodium content of 300 mg/100 g for the same product category [14]. There were no consumer awareness or education campaigns delivered under the Dialogue’s mandate during the review period.

In the latter part of 2013, the Dialogue process lapsed. The last available e-newsletter from November 2012 indicated future Dialogue activities: revisiting saturated fat targets on potato, corn and extruded snacks and savoury crackers in December 2013; further roundtable meetings with the Quick Service Restaurant (QSR) sector in early 2013; expected industry roundtables for condiments in the first half of 2013; and, agreement to target a frozen potato category. Redacted Executive Committee minutes from 28 May 2013 released under Freedom of Information provide the last available status update: saturated fat targets being progressed for savoury pies, a revised QSR Sector Action Plan to be finalised and endorsed by participating companies within 3 months, planned funding renewal, and the next e-newsletter by June 2013. The next Executive Meeting was scheduled for 19 November 2013. No evidence of any of these actions progressing was identified from any source.

### Maintenance

The Dialogue failed to report on progress in implementing Food Category Action Plans over our 2-year review period. The Dialogue’s reporting mechanism appears to have been beset with problems throughout its 6 years of operation, with many reports delayed or entirely absent (Table 3). Several Category Action Plans published on the Dialogue website (savoury pies, savoury crackers, potato/corn and extruded snacks, soups and cheese) required the Department of Health to collect and report the total amount of salt (tonnes) removed from the food supply as a result of Dialogue activities but this information, if collected, has not been made publicly available. The website also refers to additional monitoring by the National Heart Foundation in the savoury crackers, potato/corn and extruded snacks, soups and cheese categories using nutrient information and sales data. No information is publicly available on these findings.

**Table 3:** Time frames for implementation, scheduled reporting and actual reporting for targeted food categories

Food Category	Reports anticipated	Time frame for activity	Reports published or missing due Released	
Breads	6 monthly in 2010–11, then annually	May 2010- December 2013	Nov 2010	Nov 2010
			May 2011	Aug 2011 (late)
			Nov 2011	Aug 2012 (late)
			Nov 2012	Missing
			Dec 2013	Missing
Ready-to-eat breakfast cereals	6 monthly in 2010–11, then annually	May 2010–December 2013	Nov 2010	Nov 2010
			May 2011	Aug 2011 (late)
			Nov 2011	Aug 2012 (late)
			Nov 2012	Missing
			Dec 2013	Missing
Processed meats	6 monthly in 2011–12, then annually	Jan 2011- Dec 2013	Jun 2011	Nov 2011 (late)
			Jan 2012	Aug 2012 (late)
			July 2012	Nov 2012 (late)
			Jul 2013	Missing
			Dec 2013	Missing
Simmer sauces	Every 2 years	Jan 2011–Dec 2014	Dec 2012	May 2013 (late)
			Dec 2014	Missing
Soups	Annually from Feb 2012	Dec 2011–Dec 2014	Feb 2012	Missing
			Feb 2013	May 2013 (late)
			Feb 2014	Missing
			Dec 2014	Missing
Savoury Pies	6 monthly	Mar 2012–Mar 2014	Sep 2012	Missing
			Mar 2013	May 2013 (late)
			Sep 2013	Missing
			Mar 2014	Missing
Potato/Corn/extruded snacks	6 monthly for first year, then annually	Mar 2012- Mar 2014	June 2013	Missing
			Dec 2013	Missing
			Dec 2014	Missing
			Dec 2015	NA
Savoury Crackers	6 monthly for first year, then annually	Dec 2012 – Dec 2015	Jun 2013	Missing
			Dec 2013	Missing
			Dec 2014	Missing
			Dec 2015	NA
Cheese	6 monthly for first year, then annually	Mar 2013- Mar 2017	Sep 2013	Missing
			Mar 2014	Missing
			Mar 2015	NA
			Mar 2016	NA
			Mar 2017	NA
Processed Poultry	Intended, but no category action plan agreed			
Noodles	Intended, but no category action plan agreed			
Condiments	Intended, but no category action plan agreed			

## Discussion

The Dialogue has done and reported little since the time of our interim assessment in October 2013. The most likely reason for this period of inaction was a change in Australia’s Federal Government on 7 September 2013, and an apparent shift in the priority given to preventive health, highlighted by the dismantling of the Australian National Preventive Health Agency. Minutes from the Dialogue’s last known Executive Group meeting on 28 May 2013 identified $800,000 allocated for a further 2 years’ work. Instead, the freeze in activity suggests the Dialogue has been in abeyance. The most recent mention of possible Dialogue activity came during a meeting of the Economic References Committee of the Australian Senate on 11 September 2015 when government was noted to be “in the process” of getting the Dialogue to meet again [15]. This seems likely to have been a reference to the Healthy Food Partnership.

Details of the Healthy Food Partnership are still emerging, but at a high level it appears to have much in common with the Dialogue (Table 1). Membership suggests greater involvement of primary industry groups and early communiqués emphasise education activities intended to support individual behaviour change. This emphasis is concerning: although individual-level dietary interventions can benefit motivated individuals, interventions that improve the food environment and require only passive community participation have the greatest public health potential. Better food environments are central to the new National Diabetes Strategy, launched alongside the Healthy Food Partnership [16]. Food environment interventions like reformulation (particularly for sodium) are recognised as a public health ‘best-buy’ by actors including the World Health Organisation and the McKinsey Global Institute [17, 18]. With only 1.7 % of Australian health expenditure used for prevention (versus 7.0 % in New Zealand and 5.9 % in Canada [19]), this high cost-effectiveness is vital. Analysis of the UK’s salt reduction initiatives suggest they have reduced population salt intake by 15 % over 7 years, averting 6000 cardiovascular diseases deaths and saving the economy £1.5bn each year [20].

The new Healthy Food Partnership will gain from alignment with ongoing national and international initiatives to promote healthy diets. Australia has committed to accelerate national efforts to prevent and control NCDs as part of a global program led by the World Health Organisation. This includes action towards nine voluntary targets that include a 30 % reduction in salt intake, a 25 % reduction in raised blood pressure, a halt in the rise of obesity and diabetes, and a 25 % reduction in premature mortality from NCDs [21]. Australia has also adopted the Health Star Rating (HSR) System as its preferred front-of-pack labelling format and embarked upon a voluntary 5 year implementation program. An education campaign that aims to increase consumer awareness and encourage further industry participation is underway [22]. Anecdotally, the system is already driving product reformulation with Kelloggs and Nestle both recently improving the composition of iconic products to enhance their HSR score [23].

The food industry has also announced a parallel food reformulation initiative. The Australian Food and Grocery Council launched the ‘Healthier Australia Commitment’ in October 2012, with a food reformulation program designed to ‘link to existing health strategies such as the Food Health Dialogue and build on these through collaborative actions’ [24]. Members committed to reduce sodium and saturated fat by 25 % and energy by 12.5 % across entire product portfolios between 2008 and 2015. KPMG was engaged to monitor and publish annual progress reports, but with the Commitment’s end date now passed, no information is available.

There is little evidence to suggest voluntary industry-led programs of this type are effective [25]. By contrast, Australian modelling suggests mandatory approaches have potential to deliver much greater health benefits [26]. Legislative programs, such as regulating maximum sodium content for specific foods, are now being implemented in a number of overseas jurisdictions [27].

Our systematic analysis of the Dialogue points to four structural elements that will be key to the success of the new Healthy Food Partnership (Table 4). First, strong government leadership supported by adequate funding will be essential. Demonstrable lack of focus on preventive health at a federal level since late 2013 must be countered by renewed, public and unambiguous commitment to a prevention agenda informed by the global targets. Further, there must be a credible expectation that government will escalate its level of oversight or introduce more demanding measures – a ‘responsive regulatory approach’ - to accelerate action if voluntary efforts fail [25, 28]. Clear leadership and the threat of regulation are recognised as a key driver of action in the UK [20], at least until a change of government in 2015. Without this, competitive relationships between industry players participating voluntarily will result in weak and vague targets, few incentives for accountability, and no sanctions for non-performance. No such credible threat existed for the Dialogue, and failure to meet commitments held no consequences. Adverse media and corporate reputation were not realistic accountability levers when so little information about the performance and progress of companies was made publicly available.

**Table 4:** Requirements for an effective Healthy Food Partnership

Government leadership and funding
- Renewed and unambiguous public commitment from ministerial level, supported by necessary funding
- Credible expectation of implementing responsive regulatory approach where sufficient progress not demonstrated
- Charismatic ministerial representative to act as facilitative leader/’honest broker’, present at all meetings, publicly committed to outcomes, able to make ‘fair calls’ as required
Clear targets and timelines
- Focus on changing food environment, not only education or increasing physical activity
- National targets explicitly aligned to Australia’s commitments to WHO global NCD targets
- Food reformulation targets explicitly aligned with national targets for reducing dietary risks
- Incorporate existing Dialogue work, accelerate reformulation activity in additional food categories, nutrients and sectors including Quick Service Restaurants (‘fast food’)
- Feasibility determined by independent technical experts e.g. CSIRO, not industry players
- Consider adopting existing targets developed for other jurisdictions (e.g. UK)
- Complement existing Health Star Rating System
- Plan to enshrine in Food Standards Code as part of responsive regulatory approach
Control for conflict of interest
- Government to set clear Terms of Reference for involvement of different stakeholders, ensuring industry *not* involved in setting policy objectives and agenda
- Agreed, explicit governance arrangements that focus on tripartite collaboration while allowing for exercise of government authority when necessary.
- Open meetings with publically available minutes
- ‘Co-chair’ approach to working groups, ensuring equal representation of perspectives and that conflicting profit motives of industry don’t derail collaborative efforts towards the Partnership’s public health objectives
- Australian Competition and Consumer Commission appointed as independent observer
Independent monitoring and evaluation
- Independently conducted regular public reporting of progress towards agreed goals and targets
- Information available on individual company compliance with voluntary commitments
- Periodic review of Partnership’s governance arrangements in light of performance
- Publicise success, highlight and act upon failure - public communication by government, ‘shadow reporting’ by consumer and public health groups, recognition scheme administered by trusted independent group

Second, the Healthy Food Partnership must be seen as a vehicle to achieve national targets for reducing dietary risks. As a member of the United Nations General Assembly, Australia has agreed to consider setting national targets and process indicators to be met by 2025, taking into account the nine global targets [29]. Clear overarching targets provide a framework for the Healthy Food Partnership to set milestones and a schedule for reporting progress. They also provide context for accelerating establishment of individual targets for specific nutrients across a broad range of food categories.

Third, the Healthy Food Partnership will need to manage the conflicts of interest that exist for industry members and industry umbrella organizations. The health objectives of the Healthy Food Partnership are unlikely to align with the commercial goals of food companies. Industry may be engaged to deliver agreed goals but cannot be involved in setting the policy agenda. Finding effective incentives for participation by industry partners will be important, and improving the HSR score for a reformulated product is one example of how enhanced healthiness might translate into marketing outcomes. An award scheme run by a respected independent authority such as Food Standards Australia New Zealand might also incentivise competition on health grounds.

Finally, objective independent monitoring and evaluation is vital. Public reporting of progress, and of companies’ compliance with commitments is an important incentive in a voluntary environment. Accountability mechanisms provide opportunities for public communications by government and political activities by consumer and public health groups [30]. Regular reporting should be supplemented by formal, periodic review of the suitability of the partnership model, and its performance in helping to achieve national goals for reductions in dietary risk factors and related disease.

Strengths of our study include its systematic approach and the use of an established framework for assessment across the Dialogue’s 6 years of operation. Although conclusions are limited by the few objective data available on the Dialogue’s achievements, the primary inferences drawn are supported by the experiences of related national and international initiatives targeting the food supply.

## Conclusions

Australia has made little progress on diet-related disease and poor diet remains a leading cause of national disease burden. A collaborative approach to improving the food environment with a focus on reformulation remains both laudable and cost-effective. Unfortunately the Dialogue’s governance and implementation model proved both inefficient and unsustainable. Available evidence suggests the problems experienced were the result of interruptions in government commitment, leadership and funding, as well as weak incentives and accountability structures for participating companies, compounded by a lack of transparency and public reporting of performance. These design weaknesses must be corrected and combined with greater government leadership and national targets for reducing dietary risks if the new Healthy Food Partnership is to reduce Australia’s preventable and costly burden of diet-related disease.

## Abbreviations

NCD, non-communicable diseases; Dialogue, food and health dialogue; QSR, quick service restaurant

## References

[1] Australian Institute of Health and Welfare (2014). Australia’s Health 2014. Australia’s health series no. 14. Cat. no. AUS 178.

[2] World Health Organisation (2014). WHO Global status report on noncommunicable diseases 2014.

[3] Institute of Health Metrics and Evaluation. Global Burden of Disease: Australia Country Profile. 2013. Report No. Seattle: Institute of Health Metrics and Evaluation; 2013. https://www.healthdata.org/sites/default/files/files/country_profiles/GBD/ihme_gbd_country_report_australia.pdf.

[4] Whiteman DC, Webb PM, Green AC, Neale RE, Fritschi L, Bain CJ (2015). Cancers in Australia in 2010 attributable to modifiable factors: summary and conclusions. Aust N Z J Public Health.

[5] United Kingdom Department of Health. Public Health Responsibility Deal [Website]. [Accessed May 2016]. Available from: https://responsibilitydeal.dh.gov.uk/about/.

[6] New York City Health Deparment. National Sodium Reduction Initiative [Website]. [Access May 2016]. Available from: https://www1.nyc.gov/site/doh/health/health-topics/national-salt-reduction-initiative.page.

[7] Australian Government Department of Health. Healthy Food Partnership Communiqué. Canberra: Australian Government; 2015.

[8] Elliott T, Trevena H, Sacks G, Dunford E, Martin J, Webster J (2014). A systematic interim assessment of the Australian government’s *Food and Health Dialogue*. Med J Aust.

[9] Jilcott S, Ammerman A, Sommers J, Glasgow RE (2007). Applying the RE-AIM framework to assess the public health impact of policy change. Ann Behav Med.

[10] Gaglio B, Shoup JA, Glasgow RE (2013). The RE-AIM Framework: A Systematic Review of Use Over Time. Am J Public Health.

[11] Australian Government Department of Health and Ageing. Food and Health Dialogue [Website].

[12] Senator JM, Smyth N. First Assistant Secretary, Population Health Division. Community Affairs Legislation Committee Hansard. Commonwealth of Australia: Senate. 2014. p. 71-4.

[13] Trevena H, Neal B, Dunford E, Wu J (2014). An Evaluation of the Effects of the Australian Food and Health Dialogue Targets on the Sodium Content of Bread, Breakfast Cereals and Processed Meats. Nutrients.

[14] Trevena H, Dunford E, Neal B, Webster J (2014). The Australian Food and Health Dialogue–the implications of the sodium recommendation for pasta sauces. Public Health Nutr.

[15] Moore M. Public Health Association of Australia. Economic References Committee Hansard. Canberra: Commonwealth of Australia: Senate; 2015. p 18. http://parlinfo.aph.gov.au/parlInfo/download/committees/commsen/0541e715-bceb-472b-8989-7e47625ccade/toc_pdf/Economics%20References%20Committee_2015_09_11_3786_Official.pdf;fileType=application%2Fpdf#search=%22committees/commsen/0541e715-bceb-472b-8989-7e47625ccade/0002%22.

[16] Department of Health (2015). Australian National Diabetes Strategy 2016–2020.

[17] World Health Organisation (2013). Global Action Plan for the Prevention and Control of Non-communicable Diseases 2013–2020.

[18] Dobbs R, Sawers C, Thompson F, Manyika J, Woetzel J, Child P, et al. Overcoming Obesity: An Initial Economic Analysis. Discussion Papers: McKinsey Global Institute. 2014

[19] OECD. Health at a Glance 2013. OECD Indicators, OECD Publishing; 2013. http://dx.doi.org/10.1787/health_glance-2013-en.

[20] He F, Brinsden H, Macgregor G (2014). Salt reduction in the United Kingdom: a successful experiment in public health. J Hum Hypertens.

[21] World Health Organization. Sixty-sixth World Health Assembly: Follow-up to the Political Declaration of the High-level Meeting of the General Assembly on the Prevention and Control of Non-communicable Diseases. Geneva: WHA66.10; 2013. http://www.who.int/nmh/events/ncd_action_plan/en/.

[22] Australian Government. Health Star Rating Website [cited 2015 12 November]. Available from: http://healthstarrating.gov.au/internet/healthstarrating/publishing.nsf/Content/home.

[23] Han E. Uncle Tobys cuts fat, salt and sugar from muesli bars to boost health star rating. Sydney Morning Herald, Australia, 14 October 2015. http://www.smh.com.au/business/retail/uncle-tobys-cutsfat-salt-and-sugar-from-museli-bars-to-boost-health-star-rating-20151013-gk7n4j.html.

[24] Australian Food and Grocery Council. Healthier Australia Commitment. Food and Beverage Innovation. Aiming to improve the health of Australian families, Canberra, Australia. 2012. http://www.togethercounts.com.au/wpcontent/uploads/2013/02/HAC-Food-Targets.pdf. Accessed Dec 2015.

[25] Moodie R, Stuckler D, Monteiro C, Sheron N, Neal B, Thamarangsi T, et al. Profits and pandemics: prevention of harmful effects of tobacco, alcohol, and ultra-processed food and drink industries. The Lancet. 2013;381(9867):670–9. http://www.thelancet.com/journals/lancet/article/PIIS0140-6736(12)62089-3/abstract. 10.1016/S0140-6736(12)62089-3 23410611

[26] Cobiac LJ, Vos T, Veerman JL (2010). Cost-effectiveness of interventions to reduce dietary salt intake. Heart.

[27] Webster J, Trieu K, Dunford E, Hawkes C (2014). Target salt 2025: a global overview of national programs to encourage the food industry to reduce salt in foods. Nutrients.

[28] Magnusson R, Reeve B (2015). Food Reformulation, Responsive Regulation, and “Regulatory Scaffolding”: Strengthening Performance of Salt Reduction Programs in Australia and the United Kingdom. Nutrients.

[29] Outcome document of the high-level meeting of the General Assembly on the comprehensive review and assessment of the progress achieved in the prevention and control of noncommunicable diseases (Resolution A/RES/68/300). In: Sixty-eighth United Nations General Assembly documentation. New York: United Nations; 2014. Available from: http://www.un.org/depts/dhl/resguide/r68_en.shtml.

[30] Swinburn B, et al. Strengthening of accountability systems to create healthy food environments and reduce global obesity. The Lancet. 2015;385(9986):2534-45. http://www.thelancet.com/journals/lancet/article/PIIS0140-6736(14)61747-5/abstract. 10.1016/S0140-6736(14)61747-5 25703108

